# The Effect of Electrospun Polycaprolactone Nonwovens Containing Chitosan and Propolis Extracts on Fresh Pork Packaged in Linear Low-Density Polyethylene Films

**DOI:** 10.3390/foods10051110

**Published:** 2021-05-17

**Authors:** Emeli Vargas Romero, Loong-Tak Lim, Héctor Suárez Mahecha, Benjamin M. Bohrer

**Affiliations:** 1 Instituto de Ciencia y Tecnología de Alimentos, Universidad Nacional de Colombia, Bogotá 111321, Colombia; edvargasr@unal.edu.co (E.V.R.); hsuarezm@unal.edu.co (H.S.M.); 2 Department of Food Science, University of Guelph, Guelph, ON N1G-2W1, Canada; llim@uoguelph.ca; 3 Department of Animal Sciences, The Ohio State University, Columbus, OH 43210, USA

**Keywords:** active packaging, nonwovens, propolis, chitosan, polycaprolactone, meat stability, fresh pork quality

## Abstract

Fresh meat products are highly perishable and require optimal packaging conditions to maintain and potentially extend shelf-life. Recently, researchers have developed functional, active packaging systems that are capable of interacting with food products, package headspace, and/or the environment to enhance product shelf-life. Among these systems, antimicrobial/antioxidant active packaging has gained considerable interest for delaying/preventing microbial growth and deteriorative oxidation reactions. This study evaluated the effectiveness of active linear low-density polyethylene (**LLDPE**) films coated with a polycaprolactone/chitosan nonwoven (**Film 1**) or LLDPE films coated with a polycaprolactone/chitosan nonwoven fortified with Colombian propolis extract (**Film 2**). The active LLDPE films were evaluated for the preservation of fresh pork loin (longissimus dorsi) chops during refrigerated storage at 4 °C for up to 20 d. The meat samples were analyzed for pH, instrumental color, purge loss, thiobarbituric acid reactive substances (TBARS), and microbial stability (aerobic mesophilic and psychrophilic bacteria). The incorporation of the propolis-containing nonwoven layer provided antioxidant and antimicrobial properties to LLDPE film, as evidenced by improved color stability, no differences in lipid oxidation, and a delay of 4 d for the onset of bacteria growth of pork chops during the refrigerated storage period.

## 1. Introduction

Fresh meat is highly perishable and therefore requires optimal processing, packaging, and storage systems. The rapid deterioration of fresh meat quality is mainly attributed to the activation of biological reactions such as the oxidation of myoglobin to metmyoglobin, which causes meat to become brown and discolored, and lipid oxidation, which leads to off-flavors and off-aromas that are generally described as rancid [1]. Other intrinsic factors of meat such as water activity and pH favor the growth of microorganisms that cause microbial spoilage, which can lead to economic losses for the meat industry and health risks to the human population [2,3].

A number of innovative packaging strategies have been developed in recent years to extend the shelf-life of minimally processed food products. For example, researchers have developed active packaging systems that are capable of interacting with the food product, package headspace, and/or the environment to enhance product shelf-life [4,5,6,7,8,9,10]. Among these systems, antimicrobial active packaging has gained considerable interest for delaying/preventing microbial growth in the product via controlled delivery of antimicrobial agents from the package structure or add-on carrier components (e.g., label, sachet, insert) [4,11,12,13]. By virtue of their large surface area, electrospun nonwoven fibers are promising materials for the development of antimicrobial active packaging. They are versatile carriers of bioactive substances (e.g., antimicrobials, antioxidants) for controlling their release.

Polycaprolactone is a biodegradable, hydrophobic aliphatic polyester, widely used in controlled release systems of active substances [14], such as tea polyphenols, carnosic acid, and silver chloride [15,16,17]. Chitosan is a chitin-derived polysaccharide with a broad spectrum of useful attributes including antibacterial activity, film-forming capacity, and biodegradability [18]. Propolis is a natural resinous substance collected by honeybees, formed with a wide variety of chemical compounds such as polyphenols, flavonoids, and sesquiterpenes, which exhibit a wide-range of biological activities [19,20,21]. Ethanol propolis extract (PE) derived in Colombia has stood out as a natural food preservative due to its significant antioxidant, antimicrobial, and antifungal properties [22,23,24]. Moreover, the successful addition of PE as an antimicrobial has been well documented. For instance, PE has been used in the development of different antimicrobial nonwovens derived from other polymers, such as polyvinylpyrrolidone, polyurethane, poly (lactic acid), and zein. Nonwovens developed with PE have been mostly used for biomedical applications [25,26,27,28,29,30] but also for several food applications [31,32,33].

To our knowledge, linear low-density polyethylene (LLDPE) films coated with polycaprolactone nonwoven blended with either chitosan or chitosan and PE have not been reported before. Thus, the objective of the current study was to evaluate the antimicrobial and antioxidant properties of these active packaging films for the preservation of perishable meat products, using pork loin as a model system.

## 2. Materials and Methods

### 2.1. Propolis Extraction

A quantity of 60 g of raw propolis from Santander, Colombia, was mixed with 600 mL of an ether/ethanol solution (at a ratio of 60:40 *v*/*v*), placed in an ultrasound bath for 1 h, and centrifuged at 10,000 rpm for 5 min. The supernatant was filtered under vacuum conditions. The extract was then refrigerated (4 °C) for 2 h, followed by a second centrifugation at 10,000 rpm for 5 min. Finally, the extract was evaporated at 60 °C using a rotary evaporator (IKA, digital RV 10 V, Ciudad de México, CDMX, México).

The minimum inhibitory concentration of the Colombian PE was determined using the micro-dilution technique described by Bonou et al. [34]. Briefly, 317 µL of PE was dissolved in 3664 µL of Mueller Hinton broth (BD Difco, Fisher Scientific, Waltham, MA, USA) and 16 µL of Tween 80 to reach a final concentration of 80 mg/mL. Microplates (96-well) were used for the visual determination of microbial growth. Successive dilutions (two dilutions) were performed with the stock solution, and the dilutions were subsequently inoculated with 10^6^ CFU of bacteria. A positive control (bacterium + medium) and a negative control (PE + medium) were performed. The microplates were covered and incubated at 37 °C for 24 h. The bacterial strains used were *Staphylococcus aureus* ATCC 29737, *Escherichia coli* ATCC 25922 and *Salmonella enterica* subsp. *enterica serovar Enteritidis* ATCC 13076 (Thermo Fisher Scientific Culti-Loops, Waltham, MA, USA).

The total phenolic content of PE was determined according to methods described by Singleton et al. [35] and adapted by Siripatrawan and Vitchayakitti [36]. Briefly, 0.1 mL of PE was dissolved in 9.9 mL of 96% ethanol, and subsequently, 0.1 mL of the solution was added to 7 mL of Milli-Q water and 0.5 mL of Folin–Ciocalteu reagent. The mixture was allowed to equilibrate for 2 min. An aliquot of 1.5 mL of 20% sodium carbonate and 0.9 mL of Milli-Q water were added to the mixture, homogenized, and left to rest for 2 h. The reaction was read at 765 nm using a spectrophotometer (Evolution 60s, Thermo Fisher Scientific, Waltham, MA, USA). The total phenol contents in the extracts were expressed as gallic acid equivalents (mg GAE/g PE).

The method described by Moon and Shibamoto [37] was adapted to determine the antioxidant activity. A stock solution of 38.41 mg of ABTS diammonium salt (7.00 mM) and 6.63 mg of potassium persulfate (2.45 mM) was used. The absorbance was read at 734 nm using a spectrophotometer (Evolution 60s, Thermo Fisher Scientific, Waltham, MA, USA). The results were expressed as equivalent of µmol Trolox/g of PE.

### 2.2. Preparation of Solutions and Electrospinning Process

The spin dope solutions for electrospinning were comprised of polycaprolactone (MW = 80,000 Da, Sigma-Aldrich, St. Louis, Missouri, USA) and chitosan (50,000–190,000 Da, Sigma-Aldrich, St. Louis, Missouri, USA; degree of deacetylation 75–85%). Briefly, a binary solvent of formic acid (88%, #A118P-500, Fisher Scientific, Waltham, MA, USA) and glacial acetic acid (≥99.7%, #LC1010036, Fisher Scientific) at a ratio of 60:40 (*v*/*v*) was made. Then, polycaprolactone granules were added at 14% (*w*/*v*) of the binary solvent to make the polycaprolactone solution. Chitosan powder was added to the polycaprolactone solution at 8% of the polycaprolactone granules (*w*/*w*) to generate the spin dope solution for Solution 1. Propolis extract was added at 5% of the weight of polycaprolactone granules and chitosan powder (*w*/*w*) to generate Solution 2. Both solutions were mixed with a magnetic stirring bar for 18 h at 21 °C to form homogeneous spin dope solutions.

The properties of the spin dope solutions for both solutions were analyzed (in triplicate) based on methods described previously by Zaitoon and Lim [38]. Apparent viscosity of the solutions at a shear rate of 100 s^−1^ was 9.24 ± 0.01 × 10^−3^ Pa·s for Solution 1 and 7.45 ± 0.01 × 10^−3^ Pa·s for Solution 2. Electrical conductivity was 639.9 ± 5.55 µS/cm for Solution 1 and 592.9 ± 6.45 µS/cm for Solution 2. Surface tension was 56.4 ± 0.5 mN/m for Solution 1 and 52.2 ± 0.37 mN/m for Solution 2.

These spin dope solutions were electrospun into nonwovens using free surface electrospinning equipment (Nanospider, Elmarco, Czech Republic) at a constant voltage of 80 kV, a carriage speed of 50 mm/s, and an electrode-collector distance of 180 mm. Linear low-density polyethylene film (LLDPE; Glad Food Pro, Oakland, CA, USA) was used as a collection substrate for the deposition of the electrospun nonwoven, forming Film 1 (LLDPE film deposited with polycaprolactone/chitosan nonwoven; Solution 1) and Film 2 (LLDPE film deposited with polycaprolactone/chitosan nonwoven fortified with Colombian PE; Solution 2). The electrospinning process was conducted at 21 ± 2 °C and 45% RH for 35 min. The developed films were stored at 25 °C in an environmental test chamber (Sanyo, Osaka, Japan) for 24 h before further testing.

### 2.3. Pork Loin Chops

Six boneless pork loins (longissimus dorsi), with the same lot/origination number, were commercially procured from a supermarket in Guelph, Ontario, Canada. All of the pork was purchased on a common day and the common origination date suggested that all samples were of common post-mortem aging time. Nine 2.54-cm thick chops (same shape: untrimmed height and width) were cut and portioned from each loin. A total of 54 chops were weighed and randomly distributed for assignment to three different treatments as follows: (1) Treatment 1—pork chops were wrapped in a commercial LLDPE film without a functional nonwoven component (CON); (2) Treatment 2—pork chops were wrapped in Film 1; and (3) Treatment 3—pork chops were wrapped in Film 2. The wrapped and packaged pork chops were then placed inside reusable/re-sealable plastic storage bags (Ziploc; S.C. Johnson & Son, Racine, WI, USA). The bags were properly sealed and stored in a dark refrigerated room at a temperature of 4 ± 1 °C. Samples (three samples per treatment on each day) were collected for analyses on 0, 4, 8, 12, 16, and 20 days following packaging. Samples for day 0 analysis were collected at 4 h post-packaging. Following sample collection, weights for purge loss during storage, instrumental color, and pH were collected immediately. Samples were then divided into two subsamples with one subsample frozen at −20 °C for future determination of lipid oxidation and one subsample frozen at −20 °C for future microbiological analysis.

### 2.4. Purge Loss during Storage

The weight loss (WL) of chops during storage was determined according to the methodology described by Vargas and Bohrer [39]. The following equation was used:

(1) $$\%\;\mathrm{WL}=\frac{\left(W_{\mathrm{Initial}}\right)-\left(W_{\mathrm{Final}}\right)}{W_{\mathrm{Initial}}}\;\times \;100$$

where W_Initial_ was the initial weight of the pork chop, and W_Final_ was the final weight of the pork chop following the designated storage time.

### 2.5. Instrumental Color

The surface color of pork chops (film removed) was measured using a Chroma Meter CR-400 colorimeter with a 10° viewing angle and D_65_ illuminance (Konica Minolta, Osaka, Japan). The CIE L* a* b* color space was used, where L* indicated lightness of the color (L* = 0 indicates black and L* = 100 indicates white), a* indicated position between red and green (negative values indicate green while positive values indicate red), and b* indicated position between yellow and blue (negative values indicate blue and positive values indicate yellow). Total color change (ΔE) was calculated using Equation (2).

(2) $$\Delta E=\sqrt{\left({\left(L-L\prime \right)}^{2\;}+{\left(a-a\prime \right)}^{2}+{\left(b-b\prime \right)}^{2}\right)}$$

where *L*, *a*, and *b* denote the L*, a*, and b* values, respectively, of the pork loins before packaging and *L*′, *a*′, and *b*′ the L*, a*, and b* values obtained, respectively, on 4, 8, 12, 16, or 20 days of storage. The analyses were observed at three different locations for each pork chop, and averages of the three locations were reported.

### 2.6. pH Evaluation

The pH of the pork chops was measured using a portable pH meter (Hanna Instruments; HI 3779BE, Laval, Québec, QC, Canada) calibrated with pH buffer solutions of pH 4, 7, and 9. The internal pH was measured by penetration of the probe at a depth of 2.0 cm below the surface of each pork chop, and an average of three different readings was reported.

### 2.7. Lipid Oxidation

At the end of the designated storage periods, a portion of each sample was vacuum-packaged and frozen at −20 °C until further analysis for lipid oxidation. The determination of the content of thiobarbituric acid reactive substances (i.e., TBARS) was conducted to determine the concentration of malondialdehyde (MDA), which is one of several end products formed following the decomposition of lipid peroxidation products. A sample (5 g) from each chop was ground and homogenized with 1 mL of butyl hydroxytoluene and 45.5 mL of trichloroacetic acid/phosphoric acid. This was followed by filtration through a filter paper (Whatman No. 1, Fisher Scientific, Waltham, NH, USA) and addition of 5 mL of thiobarbituric acid. The solutions were incubated at 21 ± 2 °C for 22.5 h in the dark. A standard curve was created by diluting 1,1,3,3, tetraethoxy propane of 25 µM to various concentrations. The reaction obtained was read on a microplate spectrophotometer (BioTek, Winooski, VT, USA) at 530 nm. The results were reported as the quantification for mg of malondialdehyde (MDA) per kg of meat.

### 2.8. Microbial Analysis

After the designated storage periods, a portion of each pork chop was kept in the original packaging and over-packaged in a vacuum sealed package and frozen at −20 °C until the microbiological test took place. Each portion (10 g) was homogenized in 90 mL of sterile 0.1% buffered peptone water solution (Fisher Scientific, Waltham, NH, USA) using a stomacher (Seward Stomacher^®^ 400 Circulator, Lab Blender, Thomas Scientific, Swedesboro, NJ, USA) for 2 min at 300 rpm. Serial dilutions of 1:10 were prepared and homogenized using 0.1% sterile peptone water. An aliquot of 0.1 mL for each dilution was spread on PCA agar plates (Agar Plate Count, BD Difco, Fisher Scientific, Waltham, NH, USA). For aerobic mesophilic bacteria quantification, plates were incubated at 30 °C for 48 h, and for aerobic psychrophilic bacteria quantification, the plates were incubated at 4 °C for 10 d. Microbiological quantification was expressed as a logarithm of the number of colony-forming units per gram of sample (log CFU/g). Microbiological analyses were conducted in triplicate.

### 2.9. Experimental Design and Statistical Analysis

Six different pork loins were used, and from each pork loin, nine chops were obtained (*n* = 54). Three treatments and six storage days were applied to the pork chops using a completely randomized design where different loin chops were randomly assigned to treatment × day. With the completely randomized design, two to four chops from each of the six loins were randomized to each treatment.

The normality of the data was determined using PROC UNIVARIATE of SAS version 9.4 (SAS Institute, Cary, NC, USA). Subsequently, data were subjected to an ANOVA using PROC GLM of SAS with treatment (CON, Film 1 and Film 2) and storage time (0, 4, 8, 12, 16, and 20 days) as fixed effects. A Tukey–Kramer adjustment for protection of type I statistical error was applied. Statistical significance for parameters was declared at *p* < 0.05.

## 3. Results and Discussion

### 3.1. Characterization of Propolis Extracts

Raw propolis cannot be used directly as a raw material due to its high content of pollutants, which mainly consists of waxes and resins [32]. Therefore, it is necessary to extract polar and non-polar active components of propolis using an appropriate solvent. It has been reported that ethanol and ether are suitable solvents for the extraction of the main active compounds found in propolis, such as polyphenols, flavonoids, terpenoids, and fatty acids [40,41]. Using a blend of these solvents, the extraction yield calculated in the current study was 9.5%.

The antimicrobial results of PE showed a significant bactericidal effect on Gram-positive bacteria with a minimum inhibitory concentration of 0.15 mg/mL (Table 1). These results were in agreement with those reported for ethanol extracts of propolis from different geographical regions [42,43]. The PE used in the current study showed a moderate action against Gram-negative bacteria with a minimum inhibitory concentration greater than 10 mg/mL. Tosi, Ré, Ortega, and Cazzoli [31] identified a minimum inhibitory concentration of 19.2 ± 3.5 mg/mL against *E. coli* for Argentine propolis, which could be explained by the significant amounts of quercetin, galangina, caffeic acid, and chrysin found in Argentine propolis. Gutiérrez and Suárez [23] also found moderate bactericidal effects against Gram-negative bacteria for the ethanolic extracts derived from propolis from Santander, Colombia. However, Colombian propolis from other regions has shown limited activity against Gram-negative bacteria [44]. The antimicrobial action of PE depends on the interaction of its phenolic compounds with other components such as terpenes and terpenoids, both of which are bactericidal and can block cell division, limit protein synthesis [45], and destroy the cell and/or cytoplasmic wall [46].

Propolis possesses considerable antioxidant and anti-radical activity due to its phenolic compounds [47], which can interrupt lipid oxidation [48] and capture reactive oxygen species [49]. In the current study, PE contained 82.6 ± 2 mg GAE/g PE and 1186 ± 52 µmol/g PE ABTS radicals, which are indicators for total phenolic concentrations and antioxidant activity, respectively. These values were higher than those reported for PE from other Colombian regions [50,51], which may suggest that the PE from the Santander region of Colombia contained a higher concentration of active compounds, which might have contributed to the antimicrobial activity of Film 2.

### 3.2. Qualitative Observations on Composite LLDPE Films

**Figure 1:**
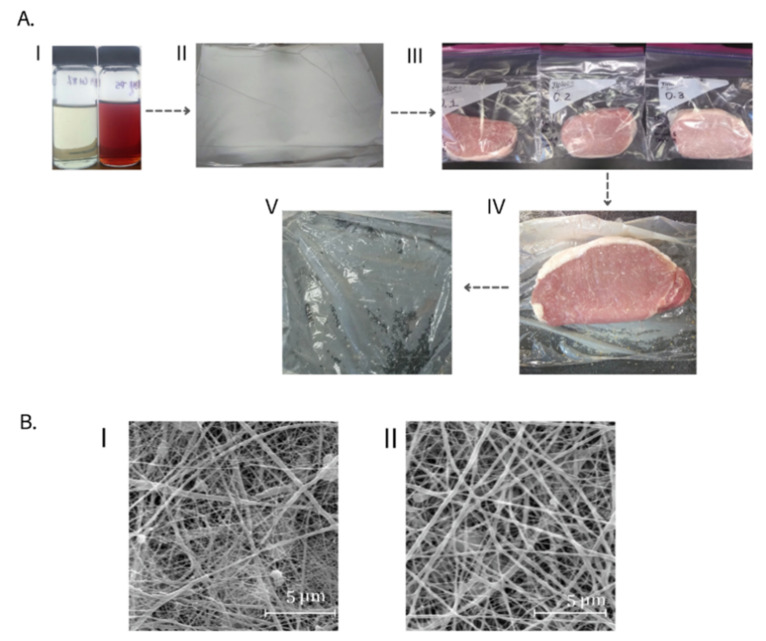
(**A**) (**I**) Spin dope solutions for electrospinning; (**II**) active nonwovens coated commercial linear low-density polyethylene (LLDPE); (**III**) treatments tested: CON (O.1)—LLDPE commercial packaging without a functional nonwoven component; Film 1 (O.2)—a commercial LLDPE film coated with a polycaprolactone/chitosan nonwoven; Film 2 (O.3)—a commercial LLDPE film coated with a polycaprolactone/chitosan nonwoven fortified with Colombian PE; (**IV**) example of coating during use; and (**V**) example of coating following use. (**B**)**.** Scanning electron micrographs of (**I**) Film 1; (**II**) Film 2.

The prepared nonwoven lined LLDPE films appeared white in color (Figure 1A, image II). However, when the nonwoven lined LLDPE film came into physical contact with the meat samples, the composite film became translucent (Figure 1A, images III and V). It is noteworthy that when the packaging was removed, the nonwoven remained adhered to the LLDPE film throughout the experiment (Figure 1A, images IV and V). The change in the white color of the coatings may have been caused by the transformation of the polycaprolactone from an initial vitreous state to an elastomeric state due to moisture transfer from the meat sample. In addition, the low differences in the refractive indices between fiber–air versus fiber–liquid of the composite within the free volume might have caused the film to become translucent [52]. As reported by Sánchez et al. [53], the moisture absorption of the polycaprolactone fibers can lower their glass transition temperature below the test temperature. The plasticization phenomenon might have also improved the flexibility of the coatings, thereby preventing them from breaking during the testing conducted in this study (i.e., pork chop application). The microstructural morphology of the two films, as observed under scanning electron microscopy, is presented in Figure 1B.

### 3.3. Meat Quality Parameters

#### 3.3.1. Purge Loss

The purge loss of the samples was affected by the storage time for all treatments (Table 2). Within each storage day, there were no differences (*p* > 0.05) among treatment for purge loss. Purge loss in CON reached the highest statistical level on day 4 of storage, while the highest statistical level for purge loss was observed on day 8 and day 16 for Film 1 and Film 2, respectively. The delay in purge loss thresholds for Film 1 and Film 2 samples may have been caused by the ultra-thin diameter of the polycaprolactone/chitosan electrospun fibers, which produces a smaller liquid-fiber contact area and subsequently increases the air-contact area [53]. On the other hand, the presence of PE in the nonwovens can reduce the hydrophilic nature of chitosan [36], which might have allowed for increased moisture retention in the pork chops.

#### 3.3.2. Instrumental Color

**Figure 2:**
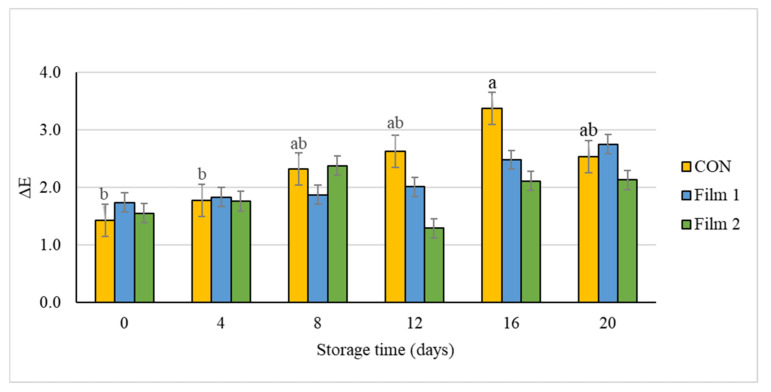
Total color difference (ΔE) of pork loin chops wrapped in active packaging for 20 days at 4 °C storage. Treatments included the following: CON—a commercial linear low-density polyethylene (LLDPE) film without a functional nonwoven component; Film 1—a commercial LLDPE film coated with a polycaprolactone/chitosan nonwoven; Film 2—commercial LLDPE film coated with a polycaprolactone/chitosan nonwoven fortified with Colombian propolis extract. ^ab^ Different letters indicate significant differences (*p* < 0.05) for the main effect of storage day for each respective treatment when a significant difference was detected. Note: no significant differences (*p* > 0.05) were detected for treatment × day, no significant differences (*p* > 0.05) were detected for storage day for Film 1 and Film 2 samples, and no significant differences (*p* > 0.05) were detected for the main effect of treatment within each day.

Changes in the color of meat during prolonged periods of storage can be indicative of lipid oxidation processes, protein oxidation processes, and microbiological contamination [54]. During the 20 days storage, there was a significant difference for instrumental lightness values among treatments on day 8, where the L* was greater (*p* < 0.05) in Film 2 samples as compared with Film 1 and CON samples. Instrumental redness (a*) value, which is an indicator of freshness for red meat, decreased during the 20 days storage period for all treatments. Samples trapped in Film 2 retained their initial redness for four days longer than the other treatments (day 0 compared to day 4), as reflected by the lower a* value for Film 2 samples on day 0. On day 12 of the storage period, pork samples wrapped in Film 1 and Film 2 had greater a* values compared with those wrapped in the CON film. This observation is indicative of the greater storage ability of samples with the coated films. For instrumental yellowness (b*) values, significant differences were observed on day 0 and day 12. On day 0, yellowness values were lower (*p* < 0.05) for Film 2 samples compared with Film 1 and CON samples. By contrast, on day 12, yellowness was greater for Film 1 samples compared with CON samples, while Film 2 samples were intermediate in value and not different compared with Film 1 and CON samples. The ΔE value is an indication of total color change over time and is often a meaningful instrumental color parameter to monitor during storage or display of meat products. The ΔE value of pork chops was affected during the storage time in CON samples in which ΔE value on day 16 was higher than on day 0 and day 4 (Figure 2). However, there were no significant changes in ΔE value for Film 1 and Film 2 samples throughout the storage period. This indicates improved color stability and thus preservation of color of Film 1 and Film 2 samples during the 20 days storage period. These results suggested that the antioxidant properties of chitosan and/or PE might have helped prevent the oxidation of the myoglobin protein and change in chemical state from myoglobin to metmyoglobin. Other studies have also confirmed that the addition of chitosan and PE either directly on samples or on edible films in meat matrices had a protective effect from oxidative deterioration [23,24,55].

#### 3.3.3. pH

**Figure 3:**
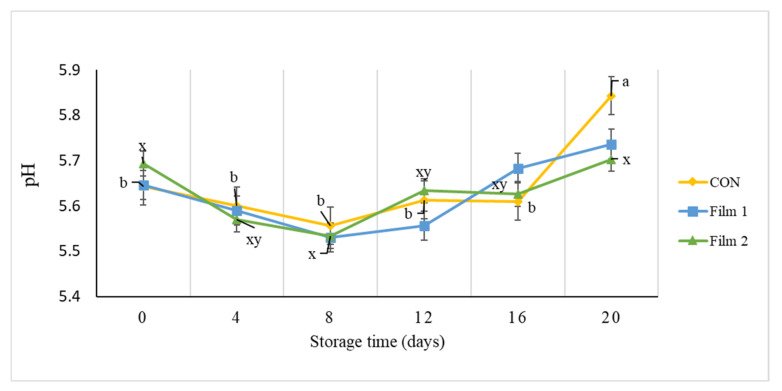
pH of pork loin chops wrapped in active packaging for 20 days at 4 °C storage. Treatments included the following: CON—a commercial linear low-density polyethylene (LLDPE) film without a functional nonwoven component; Film 1—a commercial LLDPE film coated with a polycaprolactone/chitosan nonwoven; Film 2—commercial LLDPE film coated with a polycaprolactone/chitosan nonwoven fortified with Colombian propolis extract. ^ab, xy^ Different letters indicate significant differences (*p* < 0.05) for the main effect of storage day for each respective treatment when a significant difference was detected. Note: no significant differences (*p* > 0.05) were detected for treatment × day, no significant differences (*p* > 0.05) were detected for storage day for Film 1 samples, and no significant differences (*p* > 0.05) were detected for the main effect of treatment within each day.

There was an increase (*p* < 0.05) in pH for CON samples on day 20 of the storage period compared to the other sampling days (Figure 3). No changes (*p* > 0.05) in pH were observed for the Film 1 samples during the storage period. The pH value of Film 2 samples on day 20 was higher than that on day 8, which may be indicative of the growth of lactic acid bacteria during the first 8 days of storage, due to the lag phase of PE release. These results may suggest that the chitosan and/or PE nonwoven coated LLDPE films could moderate the increase of pH for pork during the storage period. These findings lead to the speculation of two possible explanations. First, the increase in pH could be attributed to the accumulation of volatile basic nitrogen compounds from the action of microorganisms [56]. Second, endogenous meat proteases might have been involved in the change of pH during storage. It is possible for endogenous meat proteases to denature proteins during storage, causing an increase in the formation of free amino acids and peptides [57,58].

#### 3.3.4. Lipid Oxidation

Lipid oxidation values of all samples during the 20 days storage period were below the detectable rancidity threshold levels of 0.50 mg MDA/kg of meat [59]. The bioactive LLDPE films did not significantly affect (*p* > 0.05) lipid oxidation of the pork samples. Conflicting results have been reported by researchers using chitosan and PE for shelf-life extension of meat products. For example, López-Caballero et al. [60] found that gelatin–chitosan coatings were not significant in preventing lipid oxidation of cod fillets. However, other studies demonstrated significant antioxidant activity when chitosan was used as a base polymer, both in pork loins [61] and in ready-to-cook meat products [62]. Additionally, significant antioxidant activity was demonstrated when PE was used in meat and fish products [23,32]. The negligible antioxidative effect of PE observed in the current study might be due to the low concentration of the bioactives present in the pork samples that conferred a minimal protective effect against lipid oxidation. It is noteworthy that the levels of chitosan and/or PE used by other researchers, in coatings or with direct addition into the food products, were considerably higher than the levels used in the present study [23,24,55,63].

#### 3.3.5. Microbial Analysis

**Figure 4:**
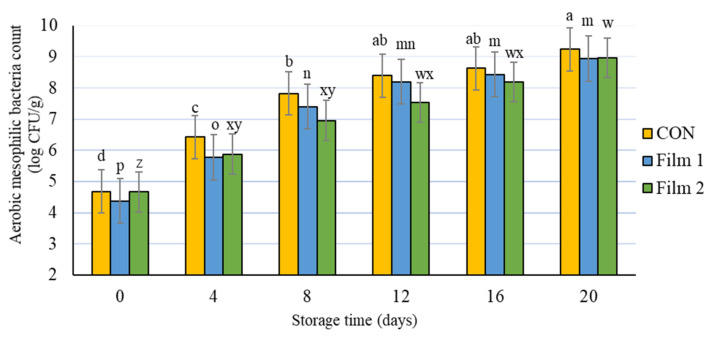
Aerobic mesophilic bacteria count of pork loin chops wrapped in active packaging for 20 days at 4 °C storage. Treatments included the following: CON—a commercial linear low-density polyethylene (LLDPE) film without a functional nonwoven component; Film 1—a commercial LLDPE film coated with a polycaprolactone/chitosan nonwoven; Film 2—commercial LLDPE film coated with a polycaprolactone/chitosan nonwoven fortified with Colombian propolis extract. ^abcd, mnop, wxyz^ Different letters indicate significant differences (*p* < 0.05) for the main effect of storage day for each respective treatment when a significant difference was detected. Note: no significant differences (*p* > 0.05) were detected for treatment × day, and no significant differences (*p* > 0.05) were detected for the main effect of treatment within each day.

**Figure 5:**
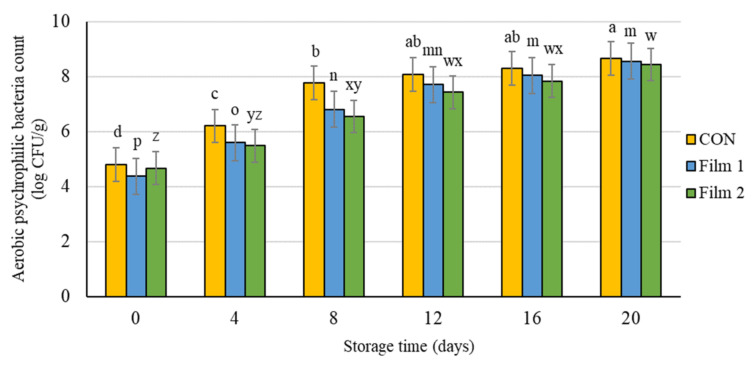
Aerobic psychrophilic bacteria count of pork loin chops wrapped in active packaging for 20 days at 4 °C storage. Treatments included the following: CON—a commercial linear low-density polyethylene (LLDPE) film without a functional nonwoven component; Film 1—a commercial LLDPE film coated with a polycaprolactone/chitosan nonwoven; Film 2—commercial LLDPE film coated with a polycaprolactone/chitosan nonwoven fortified with Colombian propolis extract. ^abcd, mnop, wxyz^ Different letters indicate significant differences (*p* < 0.05) for the main effect of storage day for each respective treatment when a significant difference was detected. Note: no significant differences (*p* > 0.05) were detected for treatment × day, and no significant differences (*p* > 0.05) were detected for the main effect of treatment within each day.

There were no differences (*p* > 0.05) among treatments at any storage day for either aerobic mesophilic bacteria count or aerobic psychrophilic bacteria count. At the beginning of the experiment, the pork samples in this study had a bacterial count for total aerobic mesophilic bacteria of 4.6 log CFU/g (Figure 4) and a bacterial count for aerobic psychrophilic bacteria of 4.7 log CFU/ g (Figure 5). Bacterial counts increased as storage time increased for both groups of bacteria and for all treatments. Although the aerobic mesophilic and aerobic psychrophilic bacteria counts were not different among the treatments, samples treated with Film 2 had delayed bacteria growth compared with the Film 1 samples and the CON samples. Specifically, aerobic mesophilic bacteria and aerobic psychrophilic bacteria counts on day 4 were similar to day 0, and the bacteria counts on day 8 were similar to day 4 for this Film 2 samples. Moreover, these values (day 0 versus day 4 and day 4 versus day 8) were different for Film 1 samples and CON samples. This observation indicated that the presence of PE in the Film 2 samples could preserve the microbiology quality of pork chops for a longer period of storage time. In fact, the bacterial counts of Film 2 samples were lower or equal to log 10^7^ CFU/g up to 12 d of storage. A value of log 10^7^ CFU/g is the microbial growth threshold level of bacteria for some regulatory purposes, which, in turn, is often used as the maximum limit that guarantees the microbiological safety of meat products. This observation is consistent with other studies that reported an improved antimicrobial effect of combining chitosan and PE [36,64] and other antimicrobials [65,66]. The findings from this study indicate that the addition of PE with chitosan in a polycaprolactone nonwoven may be a promising combination for maintaining microbial stability of meat during prolonged periods of storage.

## 4. Conclusions

The results obtained from this study suggested that the application of LLDPE film coated with a polycaprolactone/chitosan nonwoven or LLDPE film coated with a polycaprolactone/chitosan nonwoven fortified with PE had the potential to extend the quality of fresh pork during prolonged periods of refrigerated storage. Specifically, the inclusion of coated films improved color stability and microbial stability of the pork samples as evidenced by delayed color deterioration and delayed onset of aerobic mesophilic and aerobic psychrophilic bacteria growth. However, the differences between the LLDPE film coated with polycaprolactone/chitosan nonwoven and the LLDPE film coated with a polycaprolactone/chitosan nonwoven fortified with Colombian propolis extract were generally minor, in terms of magnitude, and therefore both of the films tested in this study could be recommended to the meat industry as effective active packaging materials. Future research is needed for developing a systematic understanding of the mechanistic action of chitosan and PE when introduced into a meat system. Shelf-life testing of the products packaged using the composite LLDPE films, under typical distribution and storage conditions, will also be essential for commercial applications.

## Figures and Tables

**Table 1:** Antimicrobial and antioxidant determination of Colombian propolis extract (PE) sourced from Santander, Colombia.

Minimum Inhibitory Concentration (MIC) of PE from Santander, Colombia (mg/mL)			Total Phenol Content ± SD (mg GAE/g of PE)	Antioxidant Activity ABTS TEAC ± SD (µmol Trolox/g PE)
*Staphylococcus* *aureus*	*Escherichia* *coli*	*Salmonella* *enteritidis*		
0.15	20	10	82.6 ± 2	1186 ± 52

**Table 2:** Purge loss, instrumental color, and TBARS of pork loin chops wrapped in active packaging for 20 days at 4 °C storage ^1^.

	Day 0	Day 4	Day 8	Day 12	Day 16	Day 20
Purge loss, %						
CON	0.87 ± 0.52 ^b^	3.18 ± 0.98 ^a^	3.62 ± 1.21 ^a^	4.23 ± 0.81 ^a^	4.33 ± 1.54 ^a^	4.37 ± 0.45 ^a^
Film 1	1.20 ± 0.62 ^b^	2.55 ± 0.48 ^b^	4.05 ± 1.01 ^a^	4.06 ± 0.26 ^a^	4.31 ± 0.60 ^a^	5.10 ± 1.22 ^a^
Film 2	1.57 ± 0.44 ^d^	2.37 ± 0.32 ^d^	4.34 ± 0.11 ^c^	4.54 ± 0.54 ^bc^	5.30 ± 0.41 ^ab^	5.59 ± 0.77 ^a^
Minolta L* (lightness) color, units						
CON	47.35 ± 1.93 ^b^	47.90 ± 1.85 ^ab^	49.43 ± 2.23 ^ab, y^	50.33 ± 2.75 ^a^	50.36 ± 2.02 ^a^	47.90 ± 0.85 ^ab^
Film 1	46.69 ± 2.31 ^b^	48.25 ± 2.05 ^ab^	49.16 ± 1.19 ^ab, y^	49.67 ± 1.59 ^a^	49.21 ± 1.13 ^ab^	49.00 ± 2.55 ^ab^
Film 2	45.40 ± 2.16	49.38 ± 2.97	52.20 ± 0.92 ^x^	48.35 ± 0.49	48.28 ± 1.77	49.45 ± 1.95
Minolta a* (redness) color, units						
CON	7.01 ± 1.46 ^a^	4.68 ± 1.12 ^b^	4.90 ± 1.35 ^b^	2.73 ± 0.90 ^c, y^	3.62 ± 0.56 ^bc^	4.00 ± 0.76 ^b^
Film 1	7.53 ± 1.77 ^a^	4.89 ± 0.31 ^bc^	4.82 ± 1.33 ^bc^	5.37 ± 0.93 ^b, x^	3.90 ± 0.71 ^c^	4.09 ± 0.93 ^bc^
Film 2	5.94 ± 0.43 ^a^	5.31 ± 1.03 ^ab^	3.89 ± 0.74 ^bc^	5.59 ± 0.13 ^ab, x^	4.19 ± 0.80 ^c^	4.02 ± 0.84 ^c^
Minolta b* (yellowness) color, units						
CON	4.34 ± 2.62 ^xy^	3.24 ± 0.92	3.27 ± 0.20	2.81 ± 0.24 ^y^	4.24 ± 2.04	3.44 ± 1.29
Film 1	5.07 ± 2.37 ^a, x^	3.48 ± 0.90 ^ab^	3.78 ± 1.50 ^ab^	4.28 ± 1.88 ^ab, x^	2.83 ± 0.59 ^b^	4.61 ± 1.53 ^a^
Film 2	2.61 ± 0.97 ^y^	4.29 ± 2.28	3.83 ± 1.24	3.29 ± 0.36 ^xy^	3.50 ± 1.39	3.46 ± 1.39
Thiobarbituric acid reactive substances (TBARS), mg MDA/kg meat						
CON	0.023 ± 0.001 ^ab^	0.024 ± 0.002 ^a^	0.023 ± 0.001 ^b,y^	0.025 ± 0.001 ^a^	0.024 ± 0.001 ^a^	0.024 ± 0.001 ^ab^
Film 1	0.023 ± 0.002 ^c^	0.025 ± 0.002 ^abc^	0.026 ± 0.001 ^ab,x^	0.026 ± 0.003 ^a^	0.023 ± 0.001 ^bc^	0.025 ± 0.001 ^abc^
Film 2	0.023 ± 0.002	0.024 ± 0.001	0.024 ± 0.001 ^xy^	0.026 ± 0.001	0.024 ± 0.001	0.024 ± 0.001

^abc^ For each parameter, values in the same row (day effect within each treatment) with different superscripts are different (*p* < 0.05). ^xyz^ For each parameter, values in the same column (treatment effect within each day) with different superscripts are different (*p* < 0.05). ^1^ Treatments included the following: CON—a commercial linear low-density polyethylene (LLDPE) film without a functional nonwoven component; Film 1—a commercial LLDPE film coated with a polycaprolactone/chitosan nonwoven; Film 2—commercial LLDPE film coated with a polycaprolactone/chitosan nonwoven fortified with Colombian propolis extract (PE).

## Data Availability

Data can be made available upon request.
